# First complete mitochondrial genome of the Saharan striped polecat (*Ictonyx libycus*)

**DOI:** 10.1080/23802359.2022.2141080

**Published:** 2022-11-12

**Authors:** Autumn Gray, José C. Brito, Cody W. Edwards, Henrique V. Figueiró, Klaus-Peter Koepfli

**Affiliations:** a Department of Environmental Science and Policy, George Mason University, Fairfax, VA, USA; b Smithsonian-Mason School of Conservation, George Mason University, Fairfax, VA, USA; c CIBIO, Centro de Investigação em Biodiversidade e Recursos Genéticos, InBIO Laboratório Associado, Universidade do Porto, Vairão, Portugal; d Departamento de Biologia, Faculdade de Ciências, Universidade do Porto, Porto, Portugal; e BIOPOLIS Program in Genomics, Biodiversity and Land Planning, CIBIO, Vairão, Portugal; f Department of Biology, George Mason University, Fairfax, VA, USA; g Center for Species Survival, Smithsonian’s National Zoo and Conservation Biology Institute, Virginia, USA

**Keywords:** Ictonyx libycus, mitochondrial genome, evolutionary relationships, phylogeny

## Abstract

The Saharan striped polecat (*Ictonyx libycus)* is endemic to Africa, inhabiting the edges of the Saharan Desert. Little is known about the biology or genetic status of this member of the weasel family (Mustelidae). We present the first complete mitochondrial genome of the Saharan striped polecat, assembled from data generated using a genome skimming approach. The assembled mitogenome is 16,549 bps in length and consists of 37 genes including 13 protein-coding genes, 2 rRNAs, 22 tRNAs, an origin of replication, and a control region. Phylogenetic analysis confirmed the placement of the Saharan striped polecat within the subfamily Ictonychinae.

## Introduction

The Saharan striped polecat, *Ictonyx libycus* (Hemprich and Ehrenberg 1833), also known as the Libyan striped weasel, is a member of the Mustelidae, the most speciose family within the mammalian order Carnivora. This mesocarnivore is endemic to the arid shrublands and grasslands of northern Africa, inhabiting the Mediterranean biome from Egypt to Morocco and the Sahel from Mauritania to Eritrea (Ahmim and Do Linh San 2015). Although globally listed as ‘Least Concern’ on the IUCN Red List of Threatened Species, it is locally threatened in different countries (Ahmim and Do Linh San 2015). For example, *I. libycus* is poached in Tunisia for traditional medicine, and is thus protected in that country (Ahmim and Do Linh San 2015). Like many other animal and plant species that inhabit the Sahara-Sahel, they also likely face threats from habitat loss due to human activities and conflicts (Brito et al. 2014). *Ictonyx libycus* is one of two species belonging to the genus *Ictonyx*, the other being the zorilla, *I. striatus* (Perry 1810). Here, we present the first complete mitogenome of a Saharan striped polecat to better understand its evolutionary history within the Mustelidae.

## Materials

During a field expedition in 2011 that partly focused on opportunistically collecting samples from the remains of animals that had suffered mortality by vehicles, a tissue sample from a road-killed Saharan striped polecat was collected near Timokrarin al Hamra in Rio de Oro Province, Western Sahara (24°45′25.4ʺN 14°52′05.5ʺW), preserved in ethanol and stored at room temperature and later at −80 °C. The specimen sample was deposited in the personal collection of K-P. Koepfli at the Smithsonian-Mason School of Conservation (https://smconservation.gmu.edu/people/klaus-koepfli/, Klaus-Peter Koepfli, kkoepfli@gmu.edu) under the identification number ILI_5724.

## Methods

The sample was delivered to Psomagen, Inc. (Rockville, MD) where DNA extraction, genomic library preparation, and high-throughput sequencing were performed. The Mag-Bind Blood and Tissue Kit (Omega Bio-Tek Inc., Norcross, GA) was used to extract genomic DNA. DNA concentration and quality were assayed using Picogreen and Victor X2 fluorometry (Life Technologies, Carlsbad, CA), an Agilent 4200 Tapestation (Agilent Technologies, Santa Clara, CA), and 1% gel electrophoresis. Genomic fragments of 350 base pairs (bp) were generated using a Covaris S220 Ultrasonicator (Covaris, Woburn, MA), which were used to prepare a library with the TruSeq DNA PCR-free library kit (Illumina, San Diego, CA), which was quality checked on an Agilent 4200 Tapestation and quantified using a Lightcycler qPCR assay (Roche Life Science, St. Louis, MO). The library was paired-end sequenced (2 × 150 bp) to a depth of 10x on an Illumina NovaSeq 6000 instrument, resulting in a total of 172,687,004 reads (% reads with ≥ Q30 score = 92%).

We used FastQC (Andrews 2010) to evaluate raw reads, which were then downsampled to 40 million reads using BBMap version 38.96 (Bushnell 2014). Reads were trimmed and filtered using AdapterRemoval (Lindgreen 2012) within the PALEOMIX version 1.3.6 pipeline (Schubert et al. 2014). Processed reads were imported into Geneious Prime version 2022.1 (https://www.geneious.com) and mapped to the reference mitogenome of the zorilla (GenBank accession: MW257237, published by Hassanin et al. 2021) using the Geneious mapper set to medium-low sensitivity and five iterations of fine-tuning. The mitogenome was annotated using MITOS (Bernt et al. 2013).

Multiple sequence alignment and phylogenetic analyses were performed in Geneious Prime version 2022.1. We aligned the mitogenome of the Saharan striped polecat to the mitogenomes of 20 other species of mustelids and two species of Procyonidae as outgroups (Table 1) using MAFFT version 7.450 (Katoh and Standley 2013) with the following settings: algorithm = AUTO, scoring matrix = 200 PAM/k = 2, gap open penalty = 1.53, offset value = 0.123. The alignment was visually inspected, and the control region was trimmed. The 15,563 bp alignment was then used to estimate a maximum-likelihood tree with RAxML version 8.2.11 (Stamatakis 2014) using the GTRGAMMA model of substitution and rapid hill-climbing algorithm. Nodal support was quantified with 100 bootstrap replicates.

**Table 1.** The GenBank accession numbers for the sequences used in Figure 3 with the references for the sequences.

Species	GenBank Accession Number	References
*Nasua nasua*	HM106331	(Yu et al. 2011)
*Procyon lotor*	CM027276	(Yonezawa et al. 2007)
*Taxidea taxus*	HM106330	(Yu et al. 2011)
*Mellivora capensis*	MW257239	(Hassanin et al. 2021)
*Meles meles*	MW257227	(Hassanin et al. 2021)
*Arctonyx collaris*	HM106329	(Yu et al. 2011)
*Gulo gulo*	AM711901	(Arnason et al. 2007)
*Martes americana*	HM106324	(Yu et al. 2011)
*Martes zibellina*	FJ429093	(Xu et al. 2012)
*Melogale moschata*	MW257240	(Hassanin et al. 2021)
*Neovison vison*	HM106322	(Yu et al. 2011)
*Mustela nivalis*	MW257229	(Hassanin et al. 2021)
*Mustela nigripes*	KM272752	(Zhao et al., 2016)
*Vormela peregusna*	MW013133	(Boukhdoud et al. 2021)
*Ictonyx striatus*	MW257237	(Hassanin et al. 2021)
*Poecilogale albinucha*	MW257232	(Hassanin et al. 2021)
*Galictis vittata*	MW257225	(Hassanin et al. 2021)
*Enhydra lutris*	AB291077	(Yonezawa et al. 2007)
*Lutra lutra*	LC050126	(Waku et al. 2016)
*Aonyx cinerea*	KY117535	(Mohd Salleh et al. 2017)

## Results

**Figure 1.**
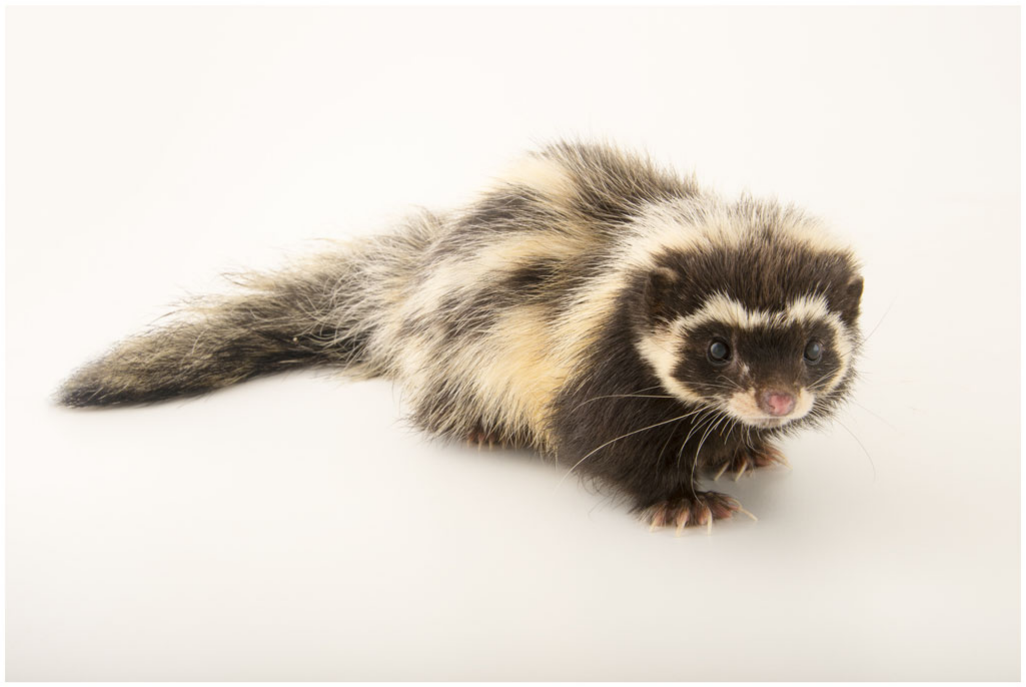
Photograph of a Saharan striped polecat, *Ictonyx libycus*. ©Joel Sartore/Photo Ark.

**Figure 2.**
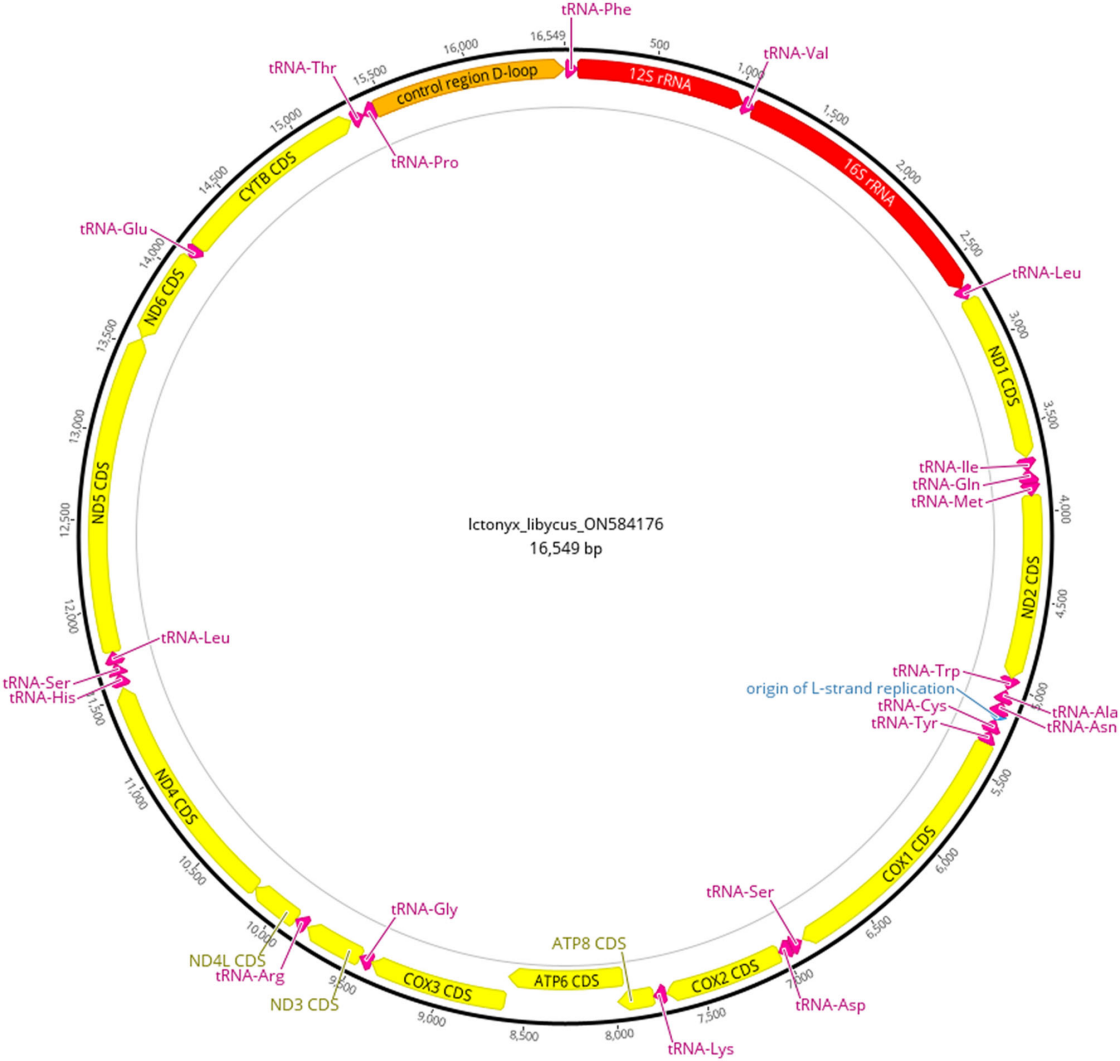
Annotated mitogenome map of the Saharan striped polecat (*Ictonyx libycus*). Colors indicate different gene types: yellow = protein-coding genes (CDS), red = rRNA genes, magenta = tRNA genes, and orange = the control region. Direction of gene arrows indicates direction of transcription. The black outer ring shows the relative nucleotide position of the components of the mitogenome.

Figure 1 shows a photograph of a Saharan striped polecat. The animal displays the typical black and white mottled fur pattern along the body and tail and the white ring around the face, which is characteristic of the species and along with a smaller body size, distinguishes it from its congener, the zorilla (*Ictonyx striatus*) (Wilson and Mittermeier 2015). We extracted a 16,549 bp consensus sequence with an average coverage of 832× from the 40 million sampled reads generated using genome skimming. Annotation of the mitogenome resulted in 13 protein-coding genes, 22 tRNAs, 2rRNAs, an origin of replication, and the control region (Figure 2).

**Figure 3.**
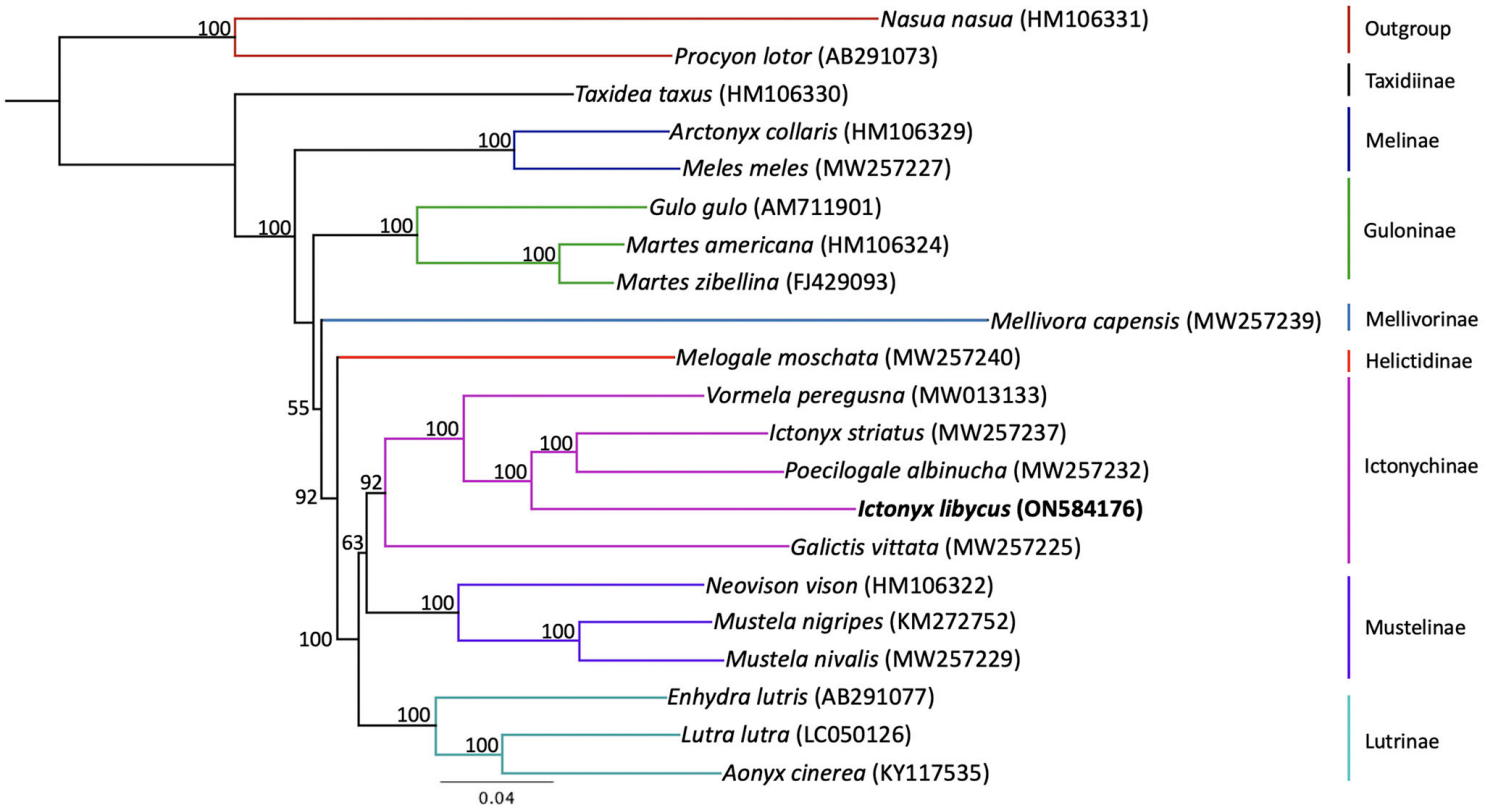
Maximum-likelihood phylogenetic tree showing the relationship of *Ictonyx libycus* within the Mustelidae. Numbers on branches are bootstrap support values (out of 100 replicates). Branch colors highlight each lineage within Mustelidae. GenBank accession numbers are included in parentheses next to the species.

Phylogenetic analysis (Figure 3) placed the Saharan striped polecat’s mitogenome with other members of the Ictonychinae and sister to a clade containing the zorilla and African striped weasel, *Poecilogale albinucha* (Gray 1864), with 100% bootstrap support. This arrangement rendered the genus *Ictonyx* paraphyletic.

## Discussion and conclusion

We assembled and annotated the first complete mitochondrial genome sequence of the Saharan striped polecat. Phylogenetic tree reconstruction using maximum likelihood revealed a topology that is congruent with previous phylogenetic analyses using multilocus datasets (Koepfli et al. 2008; Sato et al. 2012; Law et al. 2018). Our mitogenome of the Saharan striped polecat provides a resource for future phylogeographic studies of this wide-ranging and enigmatic species.

## Data Availability

The mitochondrial genome sequence is available on GenBank of NCBI at www.ncbi.nlm.nih.gov with the accession number of ON584176. The associated raw reads generated by genome skimming can be found under the BioProject ‘Lesser-known carnivores,’ accession number PRJNA847318. The associated Bio-Sample and SRA are SAMN28928423 and SRS13342623 respectively.

## References

[CIT0001] Ahmim M, Do Linh San E. 2015. Ictonyx libycus. The IUCN Red List of Threatened Species. *2015*.

[CIT0002] Andrews S. 2010. FastQC: a quality control tool for high throughput sequence data. http://www.bioinformatics.babraham.ac.uk/projects/fastqc/.

[CIT0003] Arnason U, Gullberg A, Janke A, Kullberg M. 2007. Mitogenomic analyses of caniform relationships. Mol Phylogenet Evol. 45(3):863–874. 17919938 10.1016/j.ympev.2007.06.019

[CIT0004] Bernt M, Donath A, Jühling F, Externbrink F, Florentz C, Fritzsch G, Pütz J, Middendorf M, Stadler PF. 2013. MITOS: improved de novo metazoan mitochondrial genome annotation. Mol Phylogenet Evol. 69(2):313–319. 22982435 10.1016/j.ympev.2012.08.023

[CIT0005] Boukhdoud L, Parker LD, Mcinerney NR, Saliba C, Kahale R, Cross H, Matisoo-Smith E, Maldonado JE, Bou Dagher Kharrat M. 2021. First mitochondrial genome of the marbled polecat Vormela peregusna (Carnivora, Mustelidae). Mitochondrial DNA B Resour. 6(3):1009–1011. 33796719 10.1080/23802359.2021.1894997 PMC7995864

[CIT0006] Brito JC, Godinho R, Martínez-Freiría F, Pleguezuelos JM, Rebelo H, Santos X, Vale CG, Velo-Antón G, Boratyński Z, Carvalho SB, et al. 2014. Unravelling biodiversity, evolution and threats to conservation in the Sahara-Sahel. Biol Rev Camb Philos Soc. 89(1):215–231. 23848599 10.1111/brv.12049

[CIT0007] Bushnell B. 2014. BBMap: a fast, accurate, splice-aware aligner. No. LBNL-7065E.

[CIT0009] Gray 1. E. 1864. Notice of a new species of zorilla. Proceedings of the Zoological Society of London 1864:p. 69–70.

[CIT0010] Hassanin A, Veron G, Ropiquet A, Vuuren BJv, Lécu A, Goodman SM, Haider J, Nguyen TT. 2021. Evolutionary history of Carnivora (Mammalia, Laurasiatheria) inferred from mitochondrial genomes. PLoS One. 16(2):e0240770. 33591975 10.1371/journal.pone.0240770 PMC7886153

[CIT0011] Hemprich FG, Ehrenberg CG. 1833. Symbolae Physicae, seu icones et descriptiones corporum naturalium novorum aut minus cognitorum quae ex itin eribus per Libyam Aegyptum Nubiam Dongolam Syrian Arabiam et Habessianiam publico institutis sumptu. Berlin, Germany: Signature ff. Ex Officina Academica, venditur a Mittlero.

[CIT0012] Katoh K, Standley DM. 2013. MAFFT Multiple Sequence Alignment Software Version 7: improvements in Performance and Usability. Mol Biol Evol. 30(4):772–780. 23329690 10.1093/molbev/mst010 PMC3603318

[CIT0013] Koepfli K-P, Deere KA, Slater GJ, Begg C, Begg K, Grassman L, Lucherini M, Veron G, Wayne RK. 2008. Multigene phylogeny of the Mustelidae: resolving relationships, tempo and biogeographic history of a mammalian adaptive radiation. BMC Biol. 6(1):10. 18275614 10.1186/1741-7007-6-10 PMC2276185

[CIT0014] Law CJ, Slater GJ, Mehta RS. 2018. Lineage diversity and size disparity in Musteloidea: testing patterns of adaptive radiation using molecular and fossil-based methods. Syst Biol. 67(1):127–144. 28472434 10.1093/sysbio/syx047

[CIT0015] Lindgreen S. 2012. AdapterRemoval: easy cleaning of next generation sequencing reads. BMC Res Notes. 5:337. 22748135 10.1186/1756-0500-5-337 PMC3532080

[CIT0016] Mohd Salleh F, Ramos-Madrigal J, Peñaloza F, Liu S, Mikkel-Holger SS, Riddhi PP, Martins R, Lenz D, Fickel J, Roos C, et al. 2017. An expanded mammal mitogenome dataset from Southeast Asia. Gigascience. 6(8):1–8. 10.1093/gigascience/gix053 PMC5737531 28873965

[CIT0017] Perry GC. 1810. Arcana; or, the Museum of Natural History. G. Smeeton, London, United Kingdom, single unpaged volume, signature YT (not seen, cited in Shortridge 1934).

[CIT0019] Sato JJ, Wolsan M, Prevosti FJ, D'Elía G, Begg C, Begg K, Hosoda T, Campbell KL, Suzuki H. 2012. Evolutionary and biogeographic history of weasel-like carnivorans (Musteloidea). Mol Phylogenet Evol. 63(3):745–757. 22410652 10.1016/j.ympev.2012.02.025

[CIT0020] Schubert M, Ermini L, Sarkissian CD, Jónsson H, Ginolhac A, Schaefer R, Martin MD, Fernández R, Kircher M, McCue ME, et al. 2014. Characterization of ancient and modern genomes by SNP detection and phylogenomic and metagenomic analysis using PALEOMIX. Nat Protoc. 9(5):1056–1082. 24722405 10.1038/nprot.2014.063

[CIT0021] Stamatakis A. 2014. RAxML version 8: a tool for phylogenetic analysis and post-analysis of large phylogenies. Bioinformatics. 30(9):1312–1313. 24451623 10.1093/bioinformatics/btu033 PMC3998144

[CIT0022] Waku D, Segawa T, Yonezawa T, Akiyoshi A, Ishige T, Ueda M, Ogawa H, Sasaki H, Ando M, Kohno N, et al. 2016. Evaluating the phylogenetic status of the extinct japanese otter on the basis of mitochondrial genome analysis. PLoS One. 11(3):e0149341. 26938434 10.1371/journal.pone.0149341 PMC4777564

[CIT0023] Wilson, D. E., and R. A. Mittermeier (eds.) 2015. Handbook of mammals of the world, vol. 5: monotremes and marsupials. Lynx Edicions. Barcelona: Spain, p. 799. pp. ISBN: 978-84-9655399-6

[CIT0024] Xu C, Zhang H, Ma J, Liu Z. 2012. The complete mitochondrial genome of sable, Martes zibellina. Mitochondrial DNA. 23(3):167–169. 22397380 10.3109/19401736.2012.660934

[CIT0025] Yonezawa T, Nikaido M, Kohno N, Fukumoto Y, Okada N, Hasegawa M. 2007. Molecular phylogenetic study on the origin and evolution of Mustelidae. Gene. 396(1):1–12. 17449200 10.1016/j.gene.2006.12.040

[CIT0026] Yu L, Peng D, Liu J, Luan P, Liang L, Lee H, Lee M, Ryder OA, Zhang Y. 2011. On the phylogeny of Mustelidae subfamilies: analysis of seventeen nuclear non-coding loci and mitochondrial complete genomes. BMC Evol Biol. 11(1):92. 21477367 10.1186/1471-2148-11-92 PMC3088541

[CIT0027] Zhao R-B, Zhou C-Y, Lu Z-X, Hu P, Liu J-Q, Tan W-W, Yang T-H. 2016. The complete mitochondrial genome of black-footed ferret, Mustela nigripes (Mustela, Mustelinae). Mitochondrial DNA A DNA Mapp Seq Anal. 27(3):1595–1596. 25208186 10.3109/19401736.2014.958685

